# Developing an evaluation index system for the online learning literacy of physical education teachers in China

**DOI:** 10.3389/fpsyg.2024.1243491

**Published:** 2024-02-12

**Authors:** Hengxing Tian, Mingzhu Sun, Zhihua Yin, Haohui Liu, Fangfei Li

**Affiliations:** ^1^China Basketball College, Beijing Sport University, Beijing, China; ^2^College of Physical Education and Health, East China Normal University, Shanghai, China; ^3^Department of Physical Education Teaching, Shanghai University of Engineering Science, Shanghai, China; ^4^Department of Physical Education and Military Training, Zhejiang A&F University, Hangzhou, China

**Keywords:** physical education teachers, core literacy, online learning literacy, values, essential character, key competencies

## Abstract

**Background:**

The convenience of online learning helps physical education teachers overcome geographic barriers and promotes safe, accessible, high-quality education. This three-stage study developed an evaluation index system for online learning literacy of physical education (PE) teachers (OLLPET).

**Methods:**

Using two rounds of the Delphi method and one round of the Expert ranking method, consult with 15 PE experts from universities, primary and secondary schools, and teaching-research staff to draw up, revise, and finalize an evaluation index system for OLLPET.

**Results:**

Our OLLPET evaluation index system includes three first-level indicators, seven second-level indicators and 30 third-level indicators. The first-level indicators includes online learning values (OLV), online learning essential character (OLEC), and online learning key competencies (OLKC)–with equal weighting given to OLV (0.367) and OLKC (0.367) and slightly less given to OLEC (0.267).

**Conclusion:**

The OLLPET evaluation index system is a theoretical yet practical tool that governments, schools, and teachers can use to evaluate PE teachers’ online learning literacy to improve their learning capacity in a targeted manner.

## 1 Introduction

With the globalization of education, international educational exchanges and competition have become more frequent, and countries worldwide have increased their attention on teachers’ literacy–including online literacy. At the same time, information technology (IT) penetrated all corners of our lives (Yue 2017; Rao 2018). The COVID-19 pandemic fueled a shift from purely offline learning to blended online and offline learning to make student and teacher education safe, accessible, and convenient (Comas-Quinn 2011). Physical education (PE) teachers must also reinforce their core literacy through online learning to renew teaching methods and approaches in PE classrooms (Li and Yao 2019). Although a full understanding of the online learning literacy of physical education teachers (OLLPET) could enhance the quality of PE teaching (Greenhill 2010), current research on OLLPET is limited. Therefore, we constructed an online learning literacy assessment tool applicable to PE teachers to provide scientific evaluation and feedback to advance PE teachers’ online learning.

The Strategic Plan for Teachers (2022–2025) published by the United Nations Educational, Scientific and Cultural Organization (UNESCO) proposed using multiple approaches, including information and communications technology (ICT), to enhance the quality of teaching and learning and to foster teachers’ professional development (UNESCO, 2023). Economic growth has led to an increase in global IT and changes in learning styles, expanding the popularity of online learning and making theoretical research on online learning support a hot topic. The rising need for inter-regional exchanges has driven people to seek ways of learning that transcend time and space constraints. PE teachers’ endogenous demand for online learning is the fundamental driving force behind the construction of online learning literacy assessments (Eirín-Nemiña 2015). The current level of IT use among PE teachers remains inadequate, and their online learning literacy is particularly important in the face of major public crises like the COVID-19 pandemic, which caused a global health crisis and created barriers to offline communication (Yin et al., 2020).

Online learning is highly efficient and immediate, breaking through the time and space constraints of traditional learning in ways that align with the work-related characteristics of primary and secondary school teachers. Online communication and training allow for new experiences but also place great demands on teachers’ online learning skills. Trends in the macro environment and PE teachers’ fundamental need for online learning urgently call for an in-depth study of PE teachers’ online learning literacy (Zhou 2017). Based on our search of the EBSCO and China National Knowledge Internet (CNKI) databases for articles on learning literacy, research on learning literacy has become increasingly popular since 2007. However, most relevant studies have focused on students’ learning literacy; relatively few have examined teachers’ learning literacy. Fewer still have focused on PE teachers, despite the importance of evaluating online learning for PE teachers. Thus, there is a real need for more research on OLLPET.

Research on learning literacy, teachers’ literacy, and PE teachers’ literacy has evolved from defining concepts and connotations to building assessments (Dongming et al., 2012). Constructing an evaluation index system for OLLPET has several benefits. First, it would enrich the theoretical research on PE teachers, refine research on their core literacy, lay the foundation for evaluating their learning, and provide a reference for future research on their learning literacy. Second, an assessment would help government departments and schools evaluate OLLPET while fostering PE teachers’ self-perceptions of their online learning literacy. Finally, it would help identify suggestions for PE teachers to strengthen their online learning skills and optimize their learning styles, enhancing their teaching skills and abilities. Thus, we constructed an evaluation index system for OLLPET to provide a basis for evaluating and promoting the development of OLLPET. It should be noted that the daily work of primary and secondary school PE teachers is different from that of university PE teachers, and the evaluation index system constructed in this study is mainly applicable to primary and secondary school PE teachers.

## 2 Materials and methods

**Figure 1:**
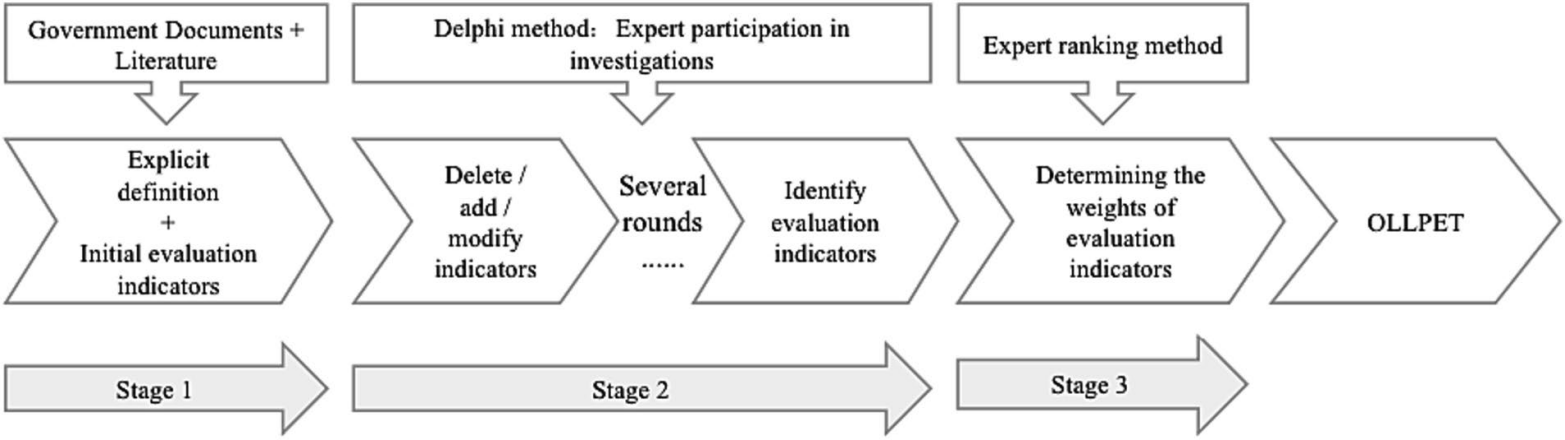
The research design of developing an evaluation index system for OLLPET.

To develop an assessment for OLLPET, we needed to divide the study into three stages. In the first stage, we established a definition of OLLPET through a literature search; we also had to select evaluation indicators for OLLPET. In the second stage, we sought expert advice using the Delphi method to identify the evaluation indicators. In the third stage, we determined the weights of the evaluation indicators via the expert-ranking method (Figure 1). The Ethics Review Committee of East China Normal University (#HR 096–2021) approved the study’s protocol.

### 2.1 Stage 1: clear definitions and initial indicators

#### 2.1.1 Defining OLLPET

This study’s core concept is the relatively new concept of OLLPET. Giving OLLPET a clear and practical definition is crucial and directly determines the research direction. A literature search revealed no standard definition for OLLPET. However, the concepts that are similar to OLLPET, online learning and learning literacy, have been well defined. Even more surprisingly, online learning for PE teachers is also mentioned in the PE teachers’ development key competency, which became the main basis for the definition of OLLPET. We integrated those to synthesize a working definition for OLLPET, described below.

#### 2.1.2 Initial screening of the evaluation indicators

At the early stage of the construction of the evaluation indicators, the evaluation indicators or frameworks in the literature were summarized, and the first draft version of the evaluation indicators for online learning literacy of PE teachers was constructed based on the frequency of key words, but the problem was that it did not have the relevance to the physical education discipline and the work characteristics of PE teachers. After a small-scale expert discussion, it was decided to decide the first-level indicators of OLLPET based on the work characteristics of PE teachers, the core literacy framework for student development issued by the Ministry of Education of China, and the definition of core literacy for PE teachers, described below. Referencing the above points, we identified online learning values (OLV), online learning essential character (OLEC), and online learning key competencies (OLKC) as the first-level indicators of OLLPET. By reviewing the literature and China’s national policies, we determined that OLLPET has three levels of indicators: The first- and second-level indicators should be concise and precise, reflecting a strong sense of framework and logic, and the third-level indicators should be detailed and specific, reflecting the characteristics of OLLPET. We selected 43 initial indicators: three first-, seven second-, and 33 third-level.

### 2.2 Stage 2: using the Delphi method to identify indicators

The Delphi method, also known as the expert survey method, uses anonymous feedback from experts to reach expert consensus and develop professional guidelines. After several rounds of consultation and feedback, the expert members’ opinions gradually converge, resulting in a collective judgment with a high accuracy rate. The Delphi method relies on rotational iteration; the investigation is incomplete until the experts reach a consensus. The Delphi method helped us ensure the objectivity and accuracy of the OLLPET evaluation indicators. We used an online survey approach, identifying the evaluation indicators through two Delphi rounds, described below.

#### 2.2.1 Selecting the experts

We needed PE teaching or research experts, preferably university research scholars, primary and secondary school PE teachers, or PE teaching-research staff. The detailed criteria for selecting the experts were these: (1) the university research scholars and teaching-research staff had to have published at least two papers on PE; (2) the primary and secondary school PE teachers had to have more than 5 years of teaching experience; and (3) all the experts had to be familiar with the core literacy research process and actively support the study.

The survey included a section for the experts’ essential information; we asked them to rate their familiarity with the indicators and provide the basis for their judgments. Each option had a corresponding score (Table 1) that we used to calculate the level of the experts’ authority as follows (Eq. 1):


(1) $$\text{Authority coefficient}=\frac{\mathrm{score}\;for\;\mathrm{familiarity}+\mathrm{score}\;for\;\mathrm{basis}\;\mathrm{of}\;\mathrm{judgement}}{2}$$

**Table 1:** Evaluation criteria for factors of experts’ authority.

Type	Option	Score
Familiarity with the indicators	Very familiar	1
	Relatively familiar	0.8
	Somewhat familiar	0.6
	Relatively unfamiliar	0.4
	Very unfamiliar	0.2
Basis for judgment	Theoretical analysis	1
	Practical experience	0.75
	Peer understanding	0.5
	Intuitive perception	0.25

The general rule is that when an expert’s authority coefficient is at or above 0.70, the expert’s opinion regarding the survey is authoritative.

#### 2.2.2 First round of the Delphi method

In a Delphi survey, the study’s background and relevant research materials need to be explained to the experts, who then judge and select the importance of each indicator. Using a five-point Likert scale, our experts classified the indicators into five levels of importance (5 = very important, 4 = important, 3 = somewhat important, 2 = unimportant, and 1 = very unimportant). We measured the indicators’ scores using three kinds of data: the average score, the standard deviation, and the coefficient of variation. By reviewing master’s and PhD theses and statistical references, we found that if an indicator score satisfied the criteria of a “mean score greater than 3.0, [a] standard deviation [of] less than 1, [and a] coefficient of variation [of] less than 0.2,” it had a high level of confidence and could be retained (Zha 2014).

#### 2.2.3 Second round of the Delphi method

After counting and analyzing the results of the first round of the survey administered to the experts, we screened and eliminated indicators that did not meet the requirements, revised indicators with reference to the experts’ comments, and prepared and distributed the second survey to the experts. After recovering and collating the results of the second survey, we needed to verify the convergence with the results of the first round. If there was convergence at this stage, the survey was closed; otherwise, it was necessary to conduct another survey until the results converged.

### 2.3 The third stage: using the expert-ranking method to determine weights

The expert-ranking method also uses surveys to elicit experts’ opinions on indicators. Experts do not need to give specific values for each indicator; they only need to rank the importance of the peer indicators. Our formula for calculating the indicator weights was as follows (Eq. 2):


(2) $$\left(a_{j}\right)=\frac{2\left[M\left(1+N\right)-Rj\right]}{MN\left(1+N\right)}$$

Where M was the number of experts participating in the survey and N was the number of indicators at the same level; aj denoted the weight of the indicator; and Rj represented the rank-sum of the jth indicator. The rank sum was the sum of the ranking numbers of m experts for a given indicator. In the case of two or more indicators of equal importance, we used the same ranking number, deferring the ranking number of the indicator that came after it (Liu et al., 2022). For example, if an expert ranked five indicators as 1, 2, 3, 3, and 5 (i.e., two indicators were tied as the third-most important), we recorded the ranking number of both tied indicators as 3.5 because (3 + 4)/2 = 3.5. This juxtaposition did not affect the calculation of the indicator weights; the sum of the weights of all indicators at each level was always equal to 1.

We performed two rounds using the Delphi method and one round using the expert-ranking method. We distributed 20 surveys in the first Delphi round and received responses from 15 experts. We sent the second Delphi round surveys only to the experts who participated in the first round; all 15 experts completed the second-round surveys and the expert-ranking method surveys. Thus, 15 experts fully participated in the two rounds of the Delphi method and the single round of the expert-ranking method (Table 2). The expert authority coefficient was 0.78, demonstrating a good level of authority across the community of experts.

**Table 2:** Basic information of experts.

Number	Age	Degree	Title	Years of work experience	Identity
E01	40–49	Ph.d	Senior teacher	20–29	University teacher
E02	40–49	Ph.d	Senior teacher	20–29	University teacher
E03	40–49	Ph.d	Sub-senior teacher	20–29	University teacher
E04	40–49	Ph.d	Sub-senior teacher	10–19	University teacher
E05	40–49	Ph.d	Sub-senior teacher	10–19	University teacher
E06	30–39	Ph.d	Sub-senior teacher	<10	University teacher
E07	30–39	Ph.d	Sub-senior teacher	<10	University teacher
E08	30–39	Ph.d	Sub-senior teacher	10–19	University teacher
E09	40–49	Ph.d	Sub-senior teacher	10–19	University teacher
E10	40–49	M.Ed	Sub-senior teacher	20–29	University teacher
E11	40–49	B.Ed	Sub-senior teacher	20–29	Teaching-research staff
E12	30–39	M.Ed	Sub-senior teacher	10–19	Primary and secondary school teacher
E13	40–49	B.Ed	Intermediate teacher	20–29	Primary and secondary school teacher
E14	30–39	B.Ed	Intermediate teacher	10–19	Primary and secondary school teacher
E15	30–39	B.Ed	Intermediate teacher	<10	Primary and secondary school teacher

## 3 Results

### 3.1 Stage 1: clearly defined and primed indicators

#### 3.1.1 Contextualizing and operationalizing the concept of OLLPET

Many scholars worldwide have defined the concept of online learning relatively clearly. Online learning is distinguished in a broad and a narrow sense. Broadly speaking, online learning refers to browsing information or digital content on the internet to gain knowledge or experience. Narrowly speaking, it means purposeful and planned learning activities that learners undertake over a fairly concentrated period to complete a specific task or goal (Jing and Li 2020). Scholars have studied learning literacy far longer than online learning. Learning literacy refers to the systematic and profound qualities that individuals develop during the learning process through repeated, ongoing practice (Qi 2013). Thus, it means the combination of competencies and qualities individuals exhibit when faced with complex learning situations. It helps individuals identify their learning needs, choose appropriate learning methods and strategies, and adjust and evaluate their learning processes.

Further sorting out research in the discipline of physical education, Fu et al. wrote that PE teachers’ core literacy was the most critical quality they could possess in the 21st century because it promotes lifelong development and teaches correct values, essential character, and essential competencies (Lingyi et al., 2019). Yin et al. (2022) constructed a theoretical model of PE teachers’ development key competency through rooted theory, and in the category of learning and reflective competence, it was clearly stated that physical education teachers should have the ability to carry out online learning related to physical education, which provided a direct theoretical basis for the definition of OLLPET. Based on this, this study views OLLPET as a subcategory of PE teachers’ development key competency.

Having clarified the notions of online learning and learning literacy, and figuring out the close connection between OLLPET and the PE teachers’ development key competency, we needed to assign a clear definition to OLLPET that was detailed and practical. The main purpose of online learning for physical education teachers is to improve their own core qualities, thus furthering the development of their students. In order to better adapt OLLPET to the job characteristics of physical education teachers, its superordinate concept had to be the PE teachers’ development key competency (Jinpeng 2017). Ultimately, we defined OLLPET as the comprehensive competencies or qualities PE teachers demonstrate by accessing learning resources and refining knowledge online. OLLPET helps PE teachers identify their learning needs, select learning strategies, and adapt and evaluate the learning process in three critical areas: values, essential character, and key competencies.

#### 3.1.2 Preliminary construction of the evaluation indicators for OLLPET

At the initial stage of the construction of the evaluation indicators, the relevant literature was reviewed with the keywords “teacher learning,” “learning literacy” and “online learning” respectively. The evaluation indexes or frameworks in the literature were summarized, and the first draft of the evaluation indexes for OLLPET was constructed based on the frequency of keywords. In the first draft, the online learning literacy of physical education teachers contains five first-level evaluation indicators, namely “learning preparation, learning methods and approaches, learning process management, learning evaluation and reflection, and learning quality.” The first draft of this version was in line with the description of the learning process, but the problem was that it was not specific to the discipline of physical education. Through careful reflection, it was found that this version of the evaluation indicators could not reflect the differences between online and traditional learning, nor could it characterize the values of PE teachers as social beings.

PE teachers’ work revolves around various teaching activities, and the logical starting point for educational work is developing students’ core literacy (Zhihua 2014). In 2018, China’s Ministry of Education defined subject core literacy as correct values, essential character, and key competencies that students gradually acquire about a given subject (Ministry of Education of the People’s republic of China 2018). Yin et al. (2022) constructed a theoretical model of core literacy for PE teachers’ development key competency through rooted theory, proposed that the core competency framework for the new era of PE teachers included three first-level indicators: correct values, essential character, and key competencies (Yin and Tian 2020; Yin et al., 2022). After referring to the work characteristics of physical education teachers, the core literacy framework for student development issued by the Ministry of Education, and the definition of PE teachers’ development key competency, and combining with some informal expert discussions in the process, this paper finally defines the “Online Learning Values, Online Learning Essential Character, and Online Learning Key Competencies” as the first-level indicators of OLLPET. Developing second-level indicators requires considering PE teachers’ ideological awareness, learning process, and practical workplace demands. OLV describe the value perspective and identification of PE teachers with online learning activities; OLEC reflects the extent to which PE teachers adhere to online learning and maintain their learning character; and OLKC reflects the knowledge, skills, and execution capabilities required for PE teachers to conduct online learning activities successfully (Kirby et al., 2010). In addition to considering the first-level indicator framework, we also referred to the results of the preliminary word frequency analyses, and finally we identified seven second-level indicators of OLPET: (1) career view (CV); (2) learning view (LV); (3) learning spirit (LS); (4) learning character (LC); (5) ability to discover learning resources (ADLR); (6) ability to plan the learning process (APLP); and (7) ability to apply learning outcomes (AALO). The first draft of the OLLPET evaluation system encompassed three first-level, seven second-level, and 30 third-level indicators. The three first-level indicators were OLV, OLEC, and OLKC, the 7 s-level indicators were CV, LV, LS, LC, ADLR, APLP and AALO. We followed these indicators with several rounds of surveys with the experts involving changes; therefore, we will not present the third level of evaluation indicators in detail here.

### 3.2 Stage 2: screening evaluation indicators using the Delphi method

#### 3.2.1 The first Delphi round

After distributing the survey and receiving the experts’ ratings of the importance of the indicators, we calculated the Average Score (AS), Standard Deviation (SD), and Coefficient of Variation (C.V) of the scores for each indicator (Table 3).

**Table 3:** The result of round 1 Delphi method.

Level	Indicator name	AS	SD	C.V
Level 1 Indicators	Online learning values	4.80	0.41	0.09
	Online learning essential character	4.67	0.62	0.13
	Online learning key competencies	4.73	0.46	0.10
Level 2 Indicators	Career view	4.53	0.52	0.11
	Learning view	4.67	0.62	0.13
	Learning spirit	4.87	0.35	0.07
	Learning character	4.73	0.59	0.13
	The ability to discover learning resources	4.80	0.41	0.09
	The ability to plan the learning process	4.67	0.49	0.10
	The ability to apply learning outcomes	4.80	0.56	0.12
Level 3 Indicators	PE teachers’ professional development teachers through online learning	4.27	0.88	0.21
	Online learning can enhance PE teachers’ teaching skills	4.53	0.52	0.11
	Online learning facilitates access to cutting-edge information in PE	4.67	0.62	0.13
	Online learning can improve the quality of PE teachers’ work	4.40	0.83	0.19
	Have a strong interest in online learning in sports	4.67	0.49	0.10
	Have a positive attitude toward online learning in sports	4.67	0.49	0.10
	Have awareness of lifelong adherence to online learning in sports	4.73	0.59	0.13
	Active adaptation to IT development in PE	4.67	0.49	0.10
	Stick to long-term online learning in sports	4.60	0.63	0.14
	Critically question online learning content in sports	4.67	0.49	0.10
	Have the courage to overcome the challenges in online learning in sports	4.67	0.49	0.10
	Reflect on the effectiveness and problems of online learning in sports	4.67	0.49	0.10
	Adhere to basic online ethics in online learning in sports	4.67	0.49	0.10
	Maintain a sense of social responsibility in online learning in sports	4.60	0.51	0.11
	Do not misuse learning resources and competencies acquired in online learning in sports	4.53	0.64	0.14
	Ability to operate a computer related to online learning in sports	4.93	0.26	0.05
	Ability to take the initiative to join a sports online learning network group	4.33	0.82	0.19
	Ability to find PE learning resources on national and international websites	4.53	0.52	0.11
	Access online learning resources for sports (e.g., micro-courses and courses of national quality)	4.80	0.41	0.09
	Explore the skills of using modern multimedia equipment (e.g., electronic whiteboards)	4.27	0.80	0.19
	Ability to explore skills using sports-related IT (e.g., pedometers, heart rate monitors, etc.)	4.13	0.92	0.22
	Be able to set reasonable online learning goals in sports based on one’s own circumstances	4.60	0.63	0.14
	Ability to select appropriate sports-related online learning content based on goals	4.67	0.72	0.16
	Ability to choose appropriate online learning tools based on the content	4.60	0.74	0.16
	Ability to effectively implement online learning plans in sports	4.60	0.63	0.14
	Ability to assess the effectiveness of one’s own online learning in sports	4.53	0.74	0.16
	Be able to apply PE online learning resources	4.80	0.41	0.09
	Ability to apply PE online learning resources in PE classroom teaching	4.93	0.26	0.05
	Ability to apply PE online learning resources in extra-curricular sports activities	4.73	0.46	0.10
	Ability to apply sports online learning resources in after-school sports training	4.73	0.46	0.10
	Ability to apply sports online learning resources in school sports competitions	4.67	0.49	0.10
	Ability to apply sport online learning resources in sports research work	4.87	0.35	0.07
	Ability to conduct sports learning activities such as lectures and exchanges through the Internet	4.60	0.63	0.14

By comparing the criteria for the retention of indicators, we found that all the first- and second-level indicators met the conditions for retention after the first round of the Delphi method; thus, we did not adjust the first- and second-level indicators. Regarding the third-level indicators, the two indicators’ coefficients of variation of scores were greater than 0.2, which did not meet the statistical requirements (Table 4). After analyzing the specific expressions of these two indicators, we found that the first one on promoting PE teachers’ professional development was too general and failed to reflect the requirements of concision and specificity; the indicator for the skill of using PE IT was more closely related to the teaching work of PE teachers and might be somewhat less relevant to online learning. After careful consideration, we decided to remove those two indicators.

**Table 4:** Third-level indicators that did not meet the statistical requirements.

Name of the indicator	AS	SD	C.V.	Decision
PE teachers’ professional development teachers through online learning	4.27	0.88	0.21	Remove indicator
Ability to explore skills using sports-related IT (e.g., pedometers, heart rate monitors, etc.)	4.13	0.92	0.22	Remove indicator

In addition to the two indicators that did not meet the statistical requirements, the experts suggested making three types of changes to the indicators: (1) strengthen the correlation between indicators at different levels and reinforce the linkage between upper- and lower-level indicators; (2) clarify logical relationships and avoid inclusion relationship between indicators at the same level; and (3) revise the language to make them clearer. The Kendall coordination coefficient W for the first survey (0.327; *p* = 0.000 < 0.01) left room for improvement since it was between 0 and 1; the larger the coefficient, the higher the expert consensus. Combining the experts’ comments, we refined the indicators once more, eventually removing four third-level indicators, adding one new third-level indicator, and revising the statements of some indicators, as detailed below.

#### 3.2.2 The second round of the Delphi method

After the first round of the Delphi method, we removed four third-level indicators (two did not meet the statistical requirements, and two were not logically related) and added one new third-level indicator. We also revised the presentation of the third-level indicators to make them more concise and specific. We compiled the revised three first-level, seven second-level, and 30 third-level indicators into a survey and sent it to the experts a second time. Analysis of the second-round Delphi data showed significant improvement in the indicators’ mean scores. Also, the standard deviations and coefficients of variation were significantly lower than in the first round, implying a significant increase in the experts’ consensus. The results of the second survey demonstrated that the mean, standard deviation, and coefficient of variation of all indicator scores met the retention requirements and had more desirable scores and stability. The Kendall coordination coefficient W for the second survey was 0.460 (*p* = 0.000 < 0.01), which was acceptable; the experts did not suggest making further changes to the indicators. Thus, we concluded the Delphi method surveys and determined the OLLPET evaluation indicators (Table 5).

**Table 5:** The result of round 2 Delphi method.

Level	Indicator name	AS	SD	C.V
Level 1 indicators	Online learning values	4.87	0.34	0.07
	Online learning essential character	4.93	0.25	0.05
	Online learning key competencies	4.93	0.25	0.05
Level 2 indicators	Career view	4.93	0.25	0.05
	Learning view	4.93	0.25	0.05
	Learning spirit	4.87	0.34	0.07
	Learning character	4.87	0.34	0.07
	Discover learning resources ability	4.87	0.34	0.07
	Plan learning process ability	4.87	0.34	0.07
	Apply learning outcomes ability	4.87	0.34	0.07
Level 3 indicators	Online learning can enhance PE teachers’ teaching skills	4.73	0.44	0.09
	Online learning facilitates access to cutting-edge information in PE	4.87	0.34	0.07
	Online learning can improve the quality of PE teachers’ work	4.80	0.40	0.08
	Have a strong interest in online learning in sports	4.73	0.44	0.09
	Have a positive attitude toward online learning in sports	4.93	0.25	0.05
	Have awareness of lifelong adherence to online learning in sports	4.87	0.34	0.07
	Active adaptation to IT development in PE	4.73	0.44	0.09
	Stick to long-term online learning in sports	4.80	0.40	0.08
	Maintain communication during online learning	4.73	0.44	0.09
	Critically question online learning content in sports	4.80	0.40	0.08
	Have the courage to overcome the challenges in online learning in sports	4.87	0.34	0.07
	Reflect on the effectiveness and problems of online learning in sports	4.87	0.34	0.07
	Adhere to basic online ethics in online learning in sports	4.80	0.40	0.08
	Maintain a sense of social responsibility in online learning in sports	4.87	0.34	0.07
	Do not misuse learning resources and competencies acquired in online learning in sports	4.73	0.57	0.12
	Ability to operate a computer related to online learning in sports	4.93	0.25	0.05
	Ability to take the initiative to join a sports online learning network group	4.53	0.62	0.14
	Ability to find PE learning resources on national and international websites	4.87	0.34	0.07
	Access online learning resources for sports (e.g., micro-courses and courses of national quality)	4.80	0.40	0.08
	Be able to set reasonable online learning goals in sports based on one’s own circumstances	4.87	0.34	0.07
	Ability to select appropriate sports-related online learning content based on goals	4.87	0.34	0.07
	Ability to choose appropriate online learning tools based on the content	4.80	0.40	0.08
	Ability to effectively implement online learning plans in sports	4.87	0.34	0.07
	Ability to assess the effectiveness of one’s own online learning in sports	4.60	0.61	0.13
	Ability to apply PE online learning resources in PE classroom teaching	4.87	0.34	0.07
	Ability to apply PE online learning resources in extra-curricular sports activities	4.47	0.62	0.14
	Ability to apply sports online learning resources in after-school sports training	4.60	0.49	0.11
	Ability to apply sports online learning resources in school sports competitions	4.80	0.40	0.08
	Ability to apply sport online learning resources in sports research work	4.80	0.40	0.08
	Ability to conduct sports learning activities such as lectures and exchanges through the Internet	4.47	0.50	0.11

### 3.3 Stage 3: determining the weights using the expert-ranking method

The survey format for the expert-ranking method was slightly different than for the Delphi method, which asked experts to judge the importance of indicators. In the expert-ranking method, the experts ranked the peer indicators in order of importance. We explained the calculation of the indicator weights earlier. Here, we provide an example of calculating indicator weights using the first-level indicator OLV. From the survey data, we can calculate the rank-sum (Rj) of OLV as 27, where the number of first-level indicators N is 3, and the number of experts M is 15:


(3) $$\left(a_{j}\right)=\frac{2\left[M\left(1+N\right)-Rj\right]}{MN\left(1+N\right)}$$

This calculation produced a weighting of 0.367 for OLV. We computed the weight of the second-level indicator CV (0.600) using the same method. However, since CV was a subordinate indicator of OLV, the final weight of CV was 0.367 × 0.600 = 0.220. We used Equation (3) to compute the weights for the remaining indicators. The Kendall coordination coefficient W for the survey of the expert-ranking method was 0.679 (*p* = 0.000 < 0.01), meaning there was strong consistency in the experts’ indicator rankings.

After calculating the indicator weights, we outlined the overall indicator framework and checked the arithmetic results. In the correct framework, the sum of the weights of all indicators at the same level should be equal to 1; otherwise, they should be recalculated. Thus, we derived the complete evaluation indicators and weights for OLLPET (Table 6).

**Table 6:** The OLLPET index system.

Level 1 indicators	Level 2 indicators	Level 3 indicators
Online learning values (0.367)	Career view (0.220)	Online learning can enhance PE teachers’ teaching skills (0.103).
		Online learning facilitates access to cutting-edge information in PE (0.051).
		Online learning can improve the quality of PE teachers’ work (0.066).
	Learning view (0.147)	Have a strong interest in online learning in sports (0.058).
		Have a positive attitude toward online learning in sports (0.040).
		Have awareness of lifelong adherence to online learning in sports (0.026).
		Active adaptation to IT development in PE (0.022).
Online learning essential character (0.267)	Learning spirit (0.166)	Stick to long-term online learning in sports (0.064).
		Maintain communication during online learning (0.039).
		Critically question online learning content in sports (0.031).
		Have the courage to overcome the challenges in online learning in sports (0.019).
		Reflect on the effectiveness and problems of online learning in sports (0.013).
	Learning character (0.101)	Adhere to basic online ethics in online learning in sports (0.047).
		Maintain a sense of social responsibility in online learning in sports (0.030).
		Do not misuse learning resources and competencies acquired in online learning in sports (0.024).
Online learning key competencies (0.367)	The ability to discover learning resources (0.179)	Ability to operate a computer related to online learning in sports (0.069).
		Ability to take the initiative to join a sports online learning network group (0.043).
		Ability to find PE learning resources on national and international websites (0.036).
		Access online learning resources for sports (e.g., micro-courses and courses of national quality) (0.031).
	The ability to plan the learning process (0.106)	Be able to set reasonable online learning goals in sports based on one’s own circumstances (0.032).
		Ability to select appropriate sports-related online learning content based on goals (0.022).
		Ability to choose appropriate online learning tools based on the content (0.023).
		Ability to effectively implement online learning plans in sports (0.013).
		Ability to assess the effectiveness of one’s own online learning in sports (0.016).
	The ability to apply learning outcomes (0.081)	Ability to apply PE online learning resources in PE classroom teaching (0.023).
		Ability to apply PE online learning resources in extra-curricular sports activities (0.016).
		Ability to apply sports online learning resources in after-school sports training (0.014).
		Ability to apply sports online learning resources in school sports competitions (0.012).
		Ability to apply sport online learning resources in sports research work (0.011).
		Ability to conduct sports learning activities such as lectures and exchanges through the Internet (0.006).

## 4 Discussion

After two Delphi rounds and one expert-ranking round, we determined the evaluation indicators and weights for OLLPET. We divided the final index system into three levels with 40 indicators that met the requirements for developing an evaluation index system with weighted indicators for the different levels.

OLV is the value content and ideals that individuals hold in relation to online learning that is positive or meets certain accepted standards; it reflects individuals’ motivations or innate drive for online learning and their awareness of online learning (Qinhua et al., 2013). In the final evaluation index system, the weight of OLV was 36.7%, reflecting the vital role of OLV in online learning. PE teachers’ online learning behaviors usually have two goals: (1) professional development and self-improvement; and (2) solutions to a specific task or problem. Thus, we included two secondary indicators under the OLV dimension: CV and LV. CV related to online learning’s contribution to professional growth or enhancing professional skills and work quality. LV related to PE teachers’ conscious (versus instinctive) self-directed learning. CV contained three third-level indicators: PE teachers’ development of their teaching skills, access to cutting-edge information in teaching, and improving teaching quality through online learning. LV contained four third-level indicators: PE teachers’ interests, attitudes, awareness, and inclinations toward online learning.

OLEC is an individual’s spirit, habit, or character that facilitates or enhances the effectiveness of their online learning; it is a stable characteristic or performance. The learning characteristics of teachers and students are universally linked and present in teaching and learning (Qingshun 2020). OLEC comprised 26.7% of the evaluation index system, which was slightly lower in weight than the other two first-level indicators but still reflected its integral role in individuals’ online learning behaviors. OLEC expressed people’s spiritual cultivation and moral character in online learning, so we divided it into two dimensions: LS and LC. LS means PE teachers’ ability to critically question and reflect over time to fulfill the strenuous demands of long-term online learning; overcome difficulties in online learning; and enhance the effectiveness of online learning. LC means PE teachers’ compliance with rules, preserving a sense of social responsibility, and promoting academic ethics in the online learning process. LS contained five third-level indicators used to demonstrate the strength of individuals’ spirit to persist in online learning and maintain communication and critical questioning while reflecting on the challenges of online learning. LC contained three third-level indicators used to demonstrate PE teachers’ sense of social responsibility, academic ethics, and the ability to apply resources properly in online learning.

OLKC encompassed the competencies individuals needed to respond to online learning situations, solve problems in online learning, and complete online learning behaviors successfully. We divided OLKC into general knowledge, information survival, and subject integration. OLKC required basic competence for applying information equipment, general learning, and the ability to learn with the support of modern information equipment (Yang and Wang 2017); its weight was 36.7% in the evaluation index system, meaning it was as crucial as OLV. We can roughly divide online learning activities according to time processes into discovering learning resources, planning the learning process, and applying learning outcomes. Thus, OLKC comprised three second-level indicators. ADLR means the capacity to find and access online resources to analyze, judge, and extract information. This determines the source and quality of online learning materials. APLP means designing and managing the online learning process, which directly determines learning outcomes. AALO means linking online learning behavior to work and society, which determines the application of learning outcomes (Dille and Røkenes 2021). ADLR comprised four third-level indicators describing an individual’s capacity to access and acquire cutting-edge online learning resources for sports and join learning communities. The APLP comprised five third-level indicators that focused on controlling learning objectives, content, plans, and outcomes. AALO comprised six third-level indicators describing PE teachers’ ability to apply online learning resources and learning outcomes in various teaching contexts, research, and lecture exchanges.

In sum, we identified the evaluation index system for OLLPET. The overall expression is OLLPET = OLV (0.367) + OLEC (0.267) + OLKC (0.367). The expression for evaluating primary indicators are OLV = CV (0.220) + LV (0.147); OLEC = LS (0.166) + LC (0.101); OLKC = ADLR (0.179) + APLP (0.106) + AALO (0.081). We also considered further applications, in terms of assessment, we can directly use the 30 third-level indicators as the question items of the assessment, and investigate the group of primary and secondary school physical education teachers. Using self-assessment or other assessment methods to get the score of each third-level indicator, and then combined with the weights of the third-level indicators to calculate the score of OLLPET. Alternatively, we can further develop the assessment scale on the basis of the indicators, and after item analysis, exploratory factor analysis, validation factor analysis and reliability test to get a perfect online learning literacy assessment scale for primary and secondary school physical education teachers, which facilitates the large-scale assessment of physical education teachers’ online learning literacy. In terms of cultivation, the government, schools and individuals can develop targeted online learning literacy cultivation measures against the evaluation index system of online learning literacy, to improve the learning behaviors of physical education teachers, promote the professional growth of physical education teachers, and help students obtain better physical education development.

## 5 Limitations

This study had some limitations. First, the primary selection of the initial indicators was based on China’s national policies and literature; thus, our assessment tool might not be generalizable to other cultures or countries without refinements. Furthermore, the use of more normative interviews or rooted theory could be considered a validation. Second, all our experts (e.g., teachers and researchers) were from Chinese cultural backgrounds. Future research should replicate the study with experts from other cultures and countries and experts from more diverse backgrounds. Third, the evaluation index system constructed in this study is mainly applicable to primary and secondary school PE teachers, although the evaluation indicators underwent a standardized screening and weighting process, actual measurement of OLLPET has yet to be carried out to verify their impact.

## 6 Conclusion

The assessment indicator system of OLLPET is a theoretical tool that can be used for practical measurements. To the best of our knowledge, this is the first study to evaluate OLLPET in China. This study’s OLLPET covered three first-level indicators—OLV, OLEC and OLKC, with equal weighting given to OLV (0.367) and OLKC (0.367) and slightly less given to OLEC (0.267); seven second-level indicators–CV (0.220), LV (0.147), LS (0.166), LC (0.101), ADLR (0.179), APLP (0.106) and AALO (0.081); and 30 third-level indicators, all weighted by level. Governments, schools, and teachers can use this system to evaluate primary and secondary school PE teachers’ online learning literacy to enhance their learning capacity in a targeted manner.

## Data availability statement

The original contributions presented in the study are included in the article/Supplementary material, further inquiries can be directed to the corresponding authors.

## Author contributions

HT, ZY, and FL: conceptualization and study design. HT and HL: data collection. HT and ZY: manuscript. MS: supervision. All authors contributed to the article and approved the submitted version.
